# Tousled kinase TLK1B mediates chromatin assembly in conjunction with Asf1 regardless of its kinase activity

**DOI:** 10.1186/1756-0500-3-68

**Published:** 2010-03-11

**Authors:** Arrigo De Benedetti

**Affiliations:** 1 Department of Biochemistry and Molecular Biology and the Feist-Weiller Cancer Center, Louisiana State University Health Sciences Center, Shreveport, LA 71130, USA

## Abstract

**Background:**

The Tousled Like Kinases (TLKs) are involved in chromatin dynamics, including DNA replication and repair, transcription, and chromosome segregation. Indeed, the first two TLK1 substrates were identified as the histone H3 and Asf1 (a histone H3/H4 chaperone), which immediately suggested a function in chromatin remodeling. However, despite the straightforward assumption that TLK1 acts simply by phosphorylating its substrates and hence modifying their activity, TLK1 also acts as a chaperone. In fact, a kinase-dead (KD) mutant of TLK1B is functional in stimulating chromatin assembly in vitro. However, subtle effects of Asf1 phosphorylation are more difficult to probe in chromatin assembly assays. Not until very recently was the Asf1 site phosphorylated by TLK1 identified. This has allowed for probing directly the functionality of a site-directed mutant of Asf1 in chromatin assembly assays.

**Findings:**

Addition of either wt or non-phosphorylatable mutant Asf1 to nuclear extract stimulates chromatin assembly on a plasmid. Similarly, TLK1B-KD stimulates chromatin assembly and it synergizes in reactions with supplemental Asf1 (wt or non-phosphorylatable mutant).

**Conclusions:**

Although the actual function of TLKs as mediators of Asf1 activity cannot be easily studied in vivo, particularly since in mammalian cells there are two TLK genes and two Asf1 genes, we were able to study specifically the stimulation of chromatin assembly in vitro. In such assays, clearly the TLK1 kinase activity was not critical, as neither a non-phosphorylatable Asf1 nor use of the TLK1B-KD impaired the stimulation of nucleosome formation.

## Background

The anti-silencing factor Asf1 was originally identified as a protein that when overexpressed derepressed the silent mating loci on chromosome III of *S. cerevisiae *[1]. These are well-characterized heterochromatic regions that, like those of telomeres and rRNA gene clusters, are transcriptionally repressed. It was later found that Asf1 is a histone H3/H4 chaperone [2] that, in conjunction with other factors like CAF1 and HIRA, can mediate both chromatin assembly and disassembly [3] during replication [4-6], transcription [7-9], and DNA repair [10-14]. It is likely that Asf1 mediates its effects by inducing localized or global chromatin remodeling, depending on the situation. Given all of these functions, it is not surprising that Asf1 is essential in mammalian cells [15] and other organisms [16], including *S. pombe *[17], but actually not in *S. cerevisiae*, although budding yeast deleted for Asf1 are sensitive to genotoxins and display elevated chromosomal instability [18]. Very recently it was found that Asf1, in conjunction with Rtt109, plays an important role in preventing replication errors at repetitive sequences in budding yeast [19]. Asf1 can be found in association with many other proteins, frequently in organism/tissue-specific fashion [20], consistent with its many molecular and developmental functions [16]. In addition to its function as a direct histone H3/H4 chaperone, Asf1 also mediates modifications of histone marks [20,21,13].

The gene *Tousled *of *Arabidopsis thaliana *encodes a protein kinase which, when mutated, results in abnormal flower development [22]. This was proposed to be linked to a replicative defect during organogenesis [23], but which may also result from failure to protect the genome from UV damage [24,12], resulting in mitotic aberrations [25-27]. Two *Tousled *genes (TLK1 and TLK2) with several splice variants were identified in mammals [28,29], and were confirmed as encoding kinases. Few physiologic substrates of *Tousled l*ike *k*inases (TLKs) have been identified, namely Asf1 [30], histone H3 [31], Aurora B [26], and more recently Rad9 in mammalian cells [32] and two mitotic kinesins in Trypanosomes [33]. This immediately suggested a function in chromatin assembly [34] during transcription [35,24], DNA repair [12,36], and condensation and segregation of chromosomes at mitosis [25]. Interestingly, all of these substrates were identified via their tight interaction with TLK, and not by classic kinase assays that usually reveal transient kinase-substrate interactions. In fact, where investigated, the association of TLK with its substrates is not ablated when a kinase-dead (KD) mutant of the protein is used [30,37,26], and in many cases it can promote functional effects in defined assays, and whether ATP is present or not [26,38,37]. This is certainly true for the binding of TLK1 with Asf1 [30,38], and in fact, the identification of hAsf1 as a substrate of TLK1 came about from a two-hybrid screen and was then confirmed with in vitro pull-downs with wt or TLK1-KD. The actual phosphorylation site was unknown at the time [30] and not identified until very recently [39]. Moreover, TLK1 can promote repair of DSBs generated with radiation [31,36] despite the fact that it is known that the actual kinase activity is inhibited due to genotoxic stress [40,41] via a DNA damage checkpoint relay [40]. This phenomenon may implicate aspects of chromatin remodeling that depend on TLK1 and Asf1 after radiation [34]. Hence, it was not very clear what could be the role of Asf1 phosphorylation by TLK, if any. Since the recent identification of the Asf1 phosphorylation site(s), in human and Drosophila, it has become possible to ascertain the potential significance of this phosphorylation. Perhaps disappointingly but maybe not surprisingly, the only effect that was reported from site-directed abrogation of the phosphorylation site in hAsf1a (and dAsf1) was an increased turnover of the protein, while hAsf1b stability was not dependent at all on phosphorylation [39]. No phenotypic effects were reported by the authors for the site-directed Asf1 mutants expressed in cell lines, and most likely there weren't any. First, Asf1 proteins are generally abundant, and even the modest (~50%) reduction reported is unlikely to result in significant effects in general aspects of chromatin dynamics. Second, at least in the case of man, there are two redundant Asf1 proteins (Asf1 and Asf1b), and only the stability of Asf1a was affected. Nonetheless, without the actual replacement of the two hAsf1 genes by site-directed mutants, it is not possible to establish the role of TLK-mediated phosphorylation in vivo. Asf1 is phosphorylated in *S. cerevisiae *(Jessica Tyler, personal communication), and of course, Asf1 replacements are easy to make in yeast, but this organism lacks TLKs. Thus, we are left with few options for establishing the role of such phosphorylation in higher organisms, in cell lines and even more importantly in transgenic animals. However, we have developed in vitro assays of chromatin assembly in mammalian extracts [12,36,14], and these assays revealed a dependence on TLK1 and Asf1a. In these assays, chromatin assembly was stimulated by the addition of wt or TLK1B-KD [14,37], but whether this effect required the presence of Asf1 or more importantly its phosphorylation was unclear. As the extract-mediated chromatin assembly in vitro requires ATP, it was not possible to dissect the specific role of Asf1 phosphorylation by TLK1. With the new information on the Asf1 phosphorylation site by TLK1 [39], it has now become possible to address the potential role of this phosphorylation at least for chromatin assembly in vitro. We found that phosphorylation of hAsf1a at S192 is not essential for nucleosome assembly.

## Findings and Discussion

### In vitro phosphorylation of Asf1 wt and mutant

**Figure 1:**
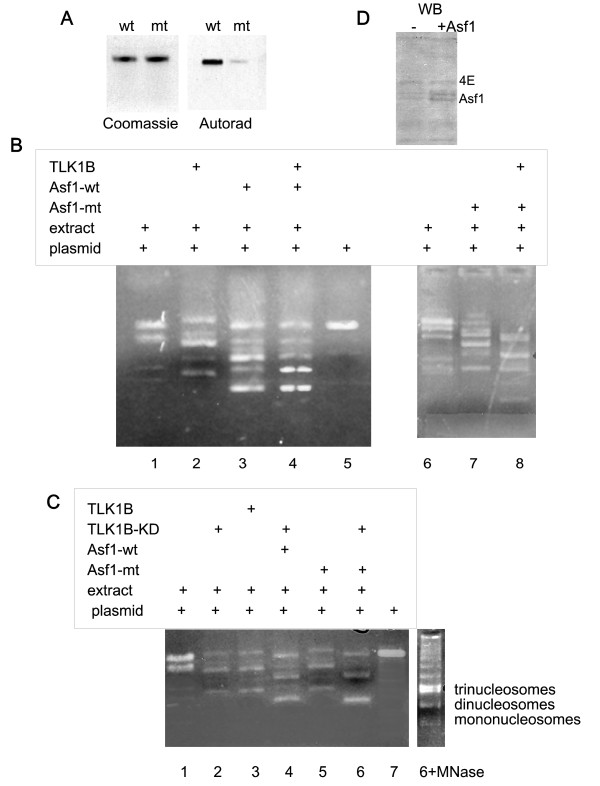
**TLK1B mediates chromatin assembly with Asf1 regardless of Asf1 phosphorylation**. **A**. In vitro phosphorylation of Asf1 wt and S192Y mutant by TLK1B. **B**. Chromatin assembly on a plasmid is stimulated equally well by Asf1 wt and mutant, and the addition of wt TLK1B further stimulates plasmid supercoiling. **C**. Chromatin assembly is stimulated by TLK1B, wt or KD, and the addition of wt or mutant Asf1 synergizes in plasmid supercoiling. **D**. Western blot for Asf1a in nuclear extract supplemented or not with recombinant Asf1a. The blot was also probed for eIF4E (4E) as a loading control.

We have expressed wt hAsf1a and the mutant, Asf1(S192Y), and carried out an in vitro phosphorylation with hTLK1B and [γ32P]ATP. We confirmed that S192 is the major phosphorylation site (Figure 1A). With this mutated Asf1, it became possible to study whether TLK1B acts as a kinase and/or as a chaperone for Asf1 in chromatin assembly.

### TLK1B stimulates chromatin assembly in vitro regardless of kinase activity and Asf1 phosphorylation

We have previously described a cell-free system in which the addition of TLK1B enhances the assembly of chromatin by an in vitro plasmid supercoiling assay, and that this depends on presence of Asf1 [12]. In this assay, Bluescript plasmid isolated from bacteria was first relaxed with wheat-germ Topoisomerase 1 (Figure 1 panel B, lane 5 and panel C, lane 7). After repurification with Geneclean, this was used as a template for the deposition of core histones in the presence of MM3MG nuclear extract and an energy mix. The extract causes the relaxed form to convert to supercoiled topoisomers of faster mobility, since some plasmid becomes bound in nucleosomes and migrates as a series of discrete forms due to a decrease in the linking number (i.e., one negative supercoil per nucleosome). The extract contains all the factors needed for chromatin assembly, including topoisomerases and core histones. The addition of Asf1, wt or mutant, resulted in stimulation of supercoiling to the same degree (Figure 1B, lanes 3 and 7). The addition of recombinant TLK1B stimulated the formation of the more highly supercoiled forms, and a similar effect was obtained for the mutant Asf1, clearly indicating that the mutant Asf1 is proficient in chromatin assembly and that TLK1B can stimulate this process in the absence of kinase activity.

To further test the idea that it is the chaperone activity of TLK1B, and not its kinase function that promotes stimulation of chromatin assembly via stimulation of Asf1, the experiment was repeated using the TLK1B-KD. Interestingly, the same activity was seen with addition of the wt or KD protein, indicating that stimulation of chromatin assembly does not depend on the kinase activity (Figure 1C). The simultaneous addition of wt or TLK1B-KD and Asf1 (wt or non-phosphorylatable mutant) synergized in the formation of more highly supercoiled plasmid forms. To confirm that the supercoiled, high-mobility forms are indeed due to formation of nucleosomes, the end-products of the reaction were subjected to MNase treatment, which resulted in the typical nucleosomal ladder (panel C, lane 6+MNase). We have previously shown that depletion of Asf1 from the nuclear extract does not preclude formation of chromatin, but that this occurs more slowly during a time course [12,14]. Hence, the addition of TLK1B and Asf1 to the extract clearly stimulates the assembly of nucleosomes, but this does not depend on TLK1B kinase activity. Rather it depends on its chaperone function. Although we should caution that endogenous Asf1a and Asf1b are obviously present in the nuclear extract, somewhat complicating the interpretation of the results, the mutant hAsf1a was added to a level ~3-fold greater than the endogenous level (western blot, panel D), and hence, should be in the optimal range as a competitor. Nonetheless, clearly these studies rely on ectopically added proteins above the endogenous level, and hence have limitations.

## Conclusions

Mutants of *Tousled *were originally identified in *A. Thaliana *based on a phenotype manifested as stunted organogenesis [22,23]. While it was assumed that the phenotype was caused by loss of the kinase activity, this was not directly determined for the specific mutants identified, and it is entirely possible that it is instead the chaperone activity of *Tousled *that is affected. In fact, it was immediately recognized that hTLK1-KD was able to bind well to hAsf1 [30]. Moreover, expression of TLK1B-KD in cells, or addition of excess mutant protein in chromatin assembly in vitro, resulted in stimulation of nucleosome formation [37,14]. Although the actual function(s) of TLKs as mediators of Asf1 activity cannot be easily studied in vivo, the recent identification of the hAsf1a phosphorylation site [39] has allowed for direct analysis of the significance of this phosphorylation in chromatin assembly in vitro. The experiments reported here suggest that the phosphorylation of Asf1 is not very significant for explaining the stimulation of TLK1B on chromatin assembly in vitro. And although there could be other activities of TLK1B (wt or KD) that may have been overlooked, such as a potential effect on histone H3 [31] or the potential formation of kinase-active dimers between wt and KD protein, the ability to detect stimulation of nucleosome formation by excess non-phosphorylatable Asf1 indicates that the role of TLK1 as a kinase for Asf1 is not very critical in these reactions. Another possible caveat is that TLK kinase activity and the phosphorylation of at least a small amount of Asf1 may still be important to initiate the process of chromatin assembly, but once started, the function of TLK1 could be less catalytic and more like a chaperone. It would be very difficult to address this caveat without the complete depletion of Asf1 and all the isoforms of TLK1 and TLK2.

We conclude that the kinase activity of TLK1B for Asf1 is not a key determinant as a modulator of Asf1 activity. Further support to the idea that TLK1 may act as a molecular chaperone for its substrates comes from its reported interaction with Aurora B in *C. elegans *[26]. TLK1 increased the activity of Aurora B in vitro in a manner that was independent of its kinase function, again suggesting that the kinase activity of TLK1, although certainly important, is insufficient to explain all its functions. Further, TLK1B-KD was proficient as the wt protein in recruiting Rad9 to a DSB in vivo, and TLK1 was found tightly associated with the chromosomal passenger complex and with two mitotic kinesins in Trypanosomes [33]. Whereas typically, most protein kinases do not bind their substrates very tightly and have only a transient interaction, TLKs bind their substrates very tightly and copurify in large complexes [41], which suggests additional activities. Also, mammalian TLK1 is a rather abundant protein, and since protein kinases have usually high turnover rates of their substrates, it seems puzzling that such a high expression would be needed based simply on a kinase role, while a function as a chaperone could explain this (recall that histone H3 and Asf1 are abundant). All of these observations suggest that TLKs are rather chaperones [14].

## Methods

Preparation of nuclear extract and chromatin assembly in vitro were as described in [12,14]. The reactions were incubated for 30 min, which is sufficient to reach equilibrated deposition of histones on the plasmid template.

Purified GST-TLK1B (wt and KD) were prepared as described in [37], and hAsf1a (CIA) was purified as described in [12]. The Asf1 unphosphorylatable mutant was generated with the oligo: 5'-gaaaact***acg***taaatgtcatgttagaatccc, changing S^192 ^and L^193 ^to Y, V, which also introduced a diagnostic SnaBI site. The mutation was confirmed by sequencing. The recombinant proteins were added at 50 nM final concentration.

## Competing interests

The author declares that he has no competing interests.

## Authors' contributions

ADB is solely responsible for this work.
